# A New Genus of Four-Legged Mites from Palms in Vietnam: The Morphology and Phylogeny of *Calventer arengii* n. g. & sp. (Eriophyoidea, Phytoptidae) †

**DOI:** 10.3390/insects16111113

**Published:** 2025-10-31

**Authors:** Philipp E. Chetverikov

**Affiliations:** Zoological Institute of Russian Academy of Sciences, Universitetskaya nab., 1, St. Petersburg 199034, Russia; pchetverikov@zin.ru

**Keywords:** gall mites, phytoparasite, COI phylogeny, chaetom, setal reduction, distribution bias

## Abstract

Eriophyoidea (Acariformes), a superfamily of obligate phytophagous and highly host-specific mites, comprises four early-diverging lineages (Pentasetacidae, Phytoptidae *s. str.*, Nalepellidae, and Eriophyidae *s.l.*) with unresolved inter-relationships. The clade Phytoptidae *s. str.* is restricted to angiosperms and includes a group of six genera associated with palms (Arecaceae) and classified in tribes Mackiellini and Phytoptini. Global distribution patterns of Phytoptidae *s. str.* are biased towards the Americas and Europe. Current knowledge of Asian palm-associated phytoptids is limited to a single record of *Borassia* from India and the new *Calventer arengii* **n. g. et sp.** (Sierraphytoptinae: Mackiellini) described here as a vagrant on the lower surface of fronds of *Arenga westerhoutii* Griff. (Arecaceae) from Vietnam. Maximum likelihood *COI* analyses did not support the monophyly of Mackiellini but revealed a sister-group relationship between *Mackiella* and *Calventer* and questioned the taxonomic position of *Borassia*. Notably, *Calventer* possesses one of the most reduced chaetoms in Phytoptidae, illustrating a recurring trend of setal loss across Eriophyoidea. Plesiomorphically, females of eriophyoids have 65 setae, with most being prone to homoplastic reduction; however, a stable subset (*h2*, *3a*, *emp*, and *ω*) is consistently retained. Excluding these “stable setae,” the remaining chaetom can be expressed as a “setal formula” incorporated into generic diagnoses for the efficient, rapid delimitation of genera.

## Simple Summary

Four-legged mites in the superfamily Eriophyoidea are a diverse group whose early evolutionary relationships are still unclear. This study focuses on a subgroup called Phytoptidae, which lives on flowering plants and includes a tribe, Mackiellini, found only on palms. In this paper, a new genus and species of phytoptid mites, *Calventer arengii*, is described. It was found on the underside of fronds (palm “leaves”) in Northeastern Vietnam. While our knowledge of phytoptids is biased toward the Americas and Europe, this finding from Asia highlights their understudied global diversity. DNA analysis showed a close relationship between the new genus and the genus *Mackiella*, known from palms in the Old World, but did not confirm that their tribe, Mackiellini, is a single evolutionary branch. Remarkably, *C. arengii* has a very reduced set of leg and body setae, illustrating a common trend of setal loss in the Eriophyoidea. We propose that by ignoring a small set of “stable” setae that all four-legged mites share, the remaining setal pattern can be used as a simple “formula” to help scientists quickly identify and classify new mite genera.

## 1. Introduction

**Figure 1:**
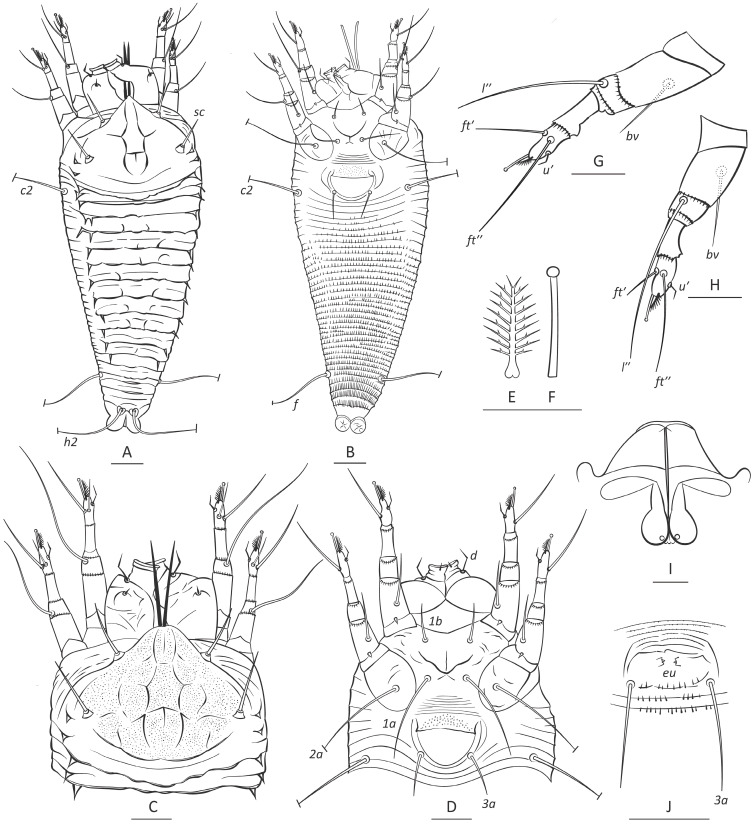
Drawings of *Calventer arengii* **n. sp.** (**A**,**B**)—entire dorsal (**A**) and ventral (**B**) female, (**C**)—prodorsal shield, dorsal gnathosoma and legs, (**D**)—ventral gnathosoma, legs and coxigenital area, (**E**)—empodium I, (**F**)—tarsal solenidion I, (**G**)—leg I, (**H**)—leg II, (**I**)—female internal genitalia, (**J**)—male genital area. Scale bar: (**A**,**B**)—15 μm; (**C**,**D**,**G**,**H**,**J**)—10 μm; (**E**,**F**,**I**)—5 μm.

Four-legged mites (superfamily Eriophyoidea) are an ancient lineage of acariform mites that evolved as obligate parasites of higher vascular plants [1]. The history of their association with plants spans about four hundred million years, with recently updated estimates placing the origin of the taxon in the Devonian (~376 Ma), when Eriophyoidea diverged from the soil-dwelling Nematalycidae and colonized early land plants [2,3,4,5,6]. The contemporary consensus in morphological taxonomy and molecular phylogenetics of Eriophyoidea implies the presence of four early-diverged lineages with incompletely resolved relationships: Pentasetacidae (associated with ancient conifer genera *Araucaria* and *Cupressus*), Phytoptidae *s. str.* (restricted to angiosperms), Nalepellidae (restricted to gymnosperms), and Eriophyidae *s. l.* (inhabiting ferns, gymnosperms and angiosperms) [7] (figure 1). Traditionally, the members of the first three lineages have been classified as a paraphyletic family, Phytoptidae *s.l.* [8], characterized by the retention of various morphological plesiomorphies (*vi*, *ve*, *φ*, and *c1*) and highly diverse anatomy of female internal genitalia. In contrast, the morphologically more advanced large clade Eriophyidae *s. l.* has been divided into families Eriophyidae *s. str.* and Diptilomiopidae, which differ in the shape and structure of the gnathosoma and possess a more uniformly structured genital apparatus [9,10,11].

While eriophyoid mites are morphologically highly specialized for feeding on plants [1] and are considered highly host-specific parasites [12,13,14], the evolution of their specificity (whether hostal, ecological, or phylogenetic) remains poorly studied. Remarkably, some eriophyoid lineages are distinctly restricted to certain genera or families of host plants, while others do not exhibit similar patterns [12]. The clade Phytoptidae *s. str.*, which is associated only with angiosperm hosts, comprises a series of genera, each restricted to one or two of three host groups: early-diverged angiosperms, monocots, and eudicots [11]. The only study focused on the molecular phylogenetics of Phytoptidae *s.str.* produced a partially resolved multigene tree, suggested no basal codivergence of phytoptids with angiosperms, and inferred paraphyly of the phytoptids associated with monocots and eudicots. This study also showed that a group of palm-inhabiting phytoptids of the tribe Mackiellini is probably paraphyletic, with two mackielline genera (*Retracrus* and *Borassia*) tending to occupy a basal position in the phytoptid tree, while the other (*Mackiella*) is nested among clades of phytoptids from eudicots [11].

The complex of phytoptids associated with palms (monocots: commelinids: Arecaceae) comprises six genera (*Acathrix* Keifer, *Borassia* Chetverikov, Craemer, *Mackiella* Keifer, *Palmiphytoptus* Navia and Flechtmann, *Propilus* Keifer, and *Retracrus* Keifer) currently classified within the tribes Phytoptini (Phytoptinae) and Mackiellini (Sierraphytoptinae) [8,15]. Most mackielline species and genera have been described from the Americas. This “New World distribution pattern” of mackiellines is surprising because palms are also very diverse in the Old World [16]. To help resolve this contradiction, I intentionally sampled palms during field trips to Northeastern Vietnam in March 2024. Among the four sampled palm species (*Arenga westerhoutii*, *Calamus tetradactylus*, *Livistona saribus*, and *Rhapis excelsa*), only one species was infested by phytoptid mites. In this paper, I describe a new mackielline genus and species, *Calventer arengii* **n. g. & sp.**, from *A. westerhoutii*, investigate the phylogenetic position of the new genus in Phytoptidae *s. str.*, and briefly discuss the evolution and biogeography of phytoptids from palms.

## 2. Materials and Methods

**Collection and morphological measurements:** Fronds of the three palm specimens of *Arenga westerhoutii* Griff. (Arecaceae) were sampled in Northeastern Vietnam in March 2024. Mites on the fronds were examined under a stereo microscope and collected using a minuten pin. They were slide-mounted in a modified Berlese medium with iodine [17] and cleared on a heating block at 90 °C for 3–5 h. The remaining mites were stored in an Eppendorf tube filled with 96% ethanol for future DNA extraction. The external morphology of the slide-mounted specimens was studied using a Leica DM2500 light microscope (LM, Wetzlar, Germany). Morphological descriptions were based on phase contrast (PC) and differential interference contrast (DIC) LM observations. All measurements are given in micrometers (µm); they represent lengths unless stated otherwise. For female descriptions, measurements are based on the holotype, with ranges (in brackets) derived from the paratypes and holotype. For males, only ranges are given. The terminology of eriophyoid morphology and the classification of Eriophyoidea follow [1] and [8], respectively. Mite drawings were sketched in pencil using a video projector [18], then scanned and finalized in Adobe Illustrator CC 2014 using a Wacom Intuos S (CTL-4100K-N) graphics tablet (Kazoto, Saitama, Japan).

**DNA extraction and sequencing:** For DNA extraction, 2 females of *Calventer arengii* **n. sp.** were separately crushed with a fine pin in a 1.5 μL drop of distilled water on a cavity well microscope slide. Each drop was pipetted into a thin-walled PCR tube with 30 μL of 6% solution of Chelex^®^ 100 Resin Bio Rad (Hercules, CA, USA) before being heated three times (5 min at 95 °C) in a thermostat with intermediate short vortexing. The solution above the Chelex^®^ granules was used as the DNA template for PCR to amplify the mitochondrial *COI* gene. For the PCR and sequencing, we applied the protocols and primers detailed by [19]. Sequences were obtained using BigDye Terminator v.3.1 chemistry (Applied Biosystems, Foster City, CA, USA) and a 3500×l Genetic Analyzer (Applied Biosystems). Trace files were checked and edited using Mega 7 [20].

**Sequence alignment and molecular phylogenetic analyses.** Molecular phylogenetic analyses of partial *COI* sequences were conducted to assess the phylogenetic position of the new genus. For this purpose, a *COI* dataset from a previous study on the phylogeny of Phytoptidae *s. str.* [11] was used. Two sequences of conifer-inhabiting nalepellid mites from the genus *Trisetacus* (KY922366 and KY922367) were used as distant outgroups. These were combined with sequences of phytoptids (MT712721–MT712756) and the new *COI* sequence of *Calventer arengii* **n. sp.** The sequences were checked for the absence of stop codons and aligned in MEGA 7 using the MUSCLE algorithm with default settings. The aligned sequences were trimmed at the 3′ and 5′ ends, resulting in a final alignment of 41 sequences with 1158 nucleotide and 386 amino acid positions. The *COI* sequences were analyzed as nucleotides, codons, and amino acids. Maximum likelihood analyses were conducted in IQ-TREE 2 [21]. Models of sequence evolution were selected using ModelFinder [22] as implemented in IQ-TREE 2, based on the Bayesian Information Criterion. The specific substitution models for each analysis were *COI* (nucleotides)—GTR+F+I+G4; *COI* (amino acids)—mtART+I+R3; and *COI* (codons)—GY+F+R5. Branch support values, generated from UFBoot (ultrafast bootstrap approximation with 10,000 bootstrap alignments, 1000 maximum iterations, and a minimum correlation coefficient of 0.99) and two single-branch tests (SH-aLRT with 10,000 replicates and the approximate Bayes test), were labeled on the ML trees. Trees were visualized in FigTree v1.4.3 (https://github.com/rambaut/figtree/releases/tag/v1.4.3 (accessed on 1 October 2025)).

## 3. Results

### 3.1. Systematics

**Subfamily Sierraphytoptinae Keifer 1944**

**Diagnosis.** Phytoptid mites *sensu* Amrine et al. [8] with four (paired *ve* and *sc*) or two (when *sc* is absent) prodorsal shield setae and dorso-ventrally differentiated opisthosomal annuli; associated with angiosperms.

**Tribe Mackiellini Newkirk and Keifer 1971**

**Diagnosis.** Opisthosomal seta *c1* absent; tibial solenidion *φ* I present or absent; prodorsal shield setae *ve* present, *sc* present or absent; associated with palms (Arecaceae).

**Genus *Calventer* n. g.**

**Diagnosis.** Four setae present on prodorsal shield: paired *ve* and *sc*. Tubercles of *ve* situated below anterolateral margin of prodorsal shield; *ve* directed up and anterolaterad. Tubercles of *sc* situated ahead of rear margin of prodorsal shield; *sc* directed up and divergently anteriad. Subtriangular frontal lobe of prodorsal shield present. Opisthosomal annuli dorso-ventrally differentiated into broader dorsal annuli and narrower ventral annuli. Dorsal opisthosomal annuli form two lateral longitudinal ridges. Tibial setae *l’* I, tibial solenidion *φ* I, and opisthosomal setae *c1*, *d*, *e* and *h1* absent. Tarsal setae *u’* and pedipalp setae *d* (*ppd*) angled. Empodium simple (undivided).

**Differential diagnosis**. Contrary to the four currently recognized genera of the tribe Mackiellini (*Mackiella* Keifer 1939, *Palmiphytoptus* Navia and Flechtmann 2002, *Propilus* Keifer 1975, and *Retracrus* Keifer 1965), the new genus possesses a remarkably reduced chaetom of ventral opisthosoma: opisthosomal setae *d* and *e* are absent in *Calventer* **n. g.** but present in other mackielline genera. *Calventer* **n. g.** is similar to *Propilus* in that in both genera tibial setae *l’* I and solenidion *φ* I are missing. However, in *Propilus,* only paired setae *ve* are present on the prodorsal shield (*sc* missing), while *Calventer* **n. g.** retains two pairs of prodorsal shield setae (paired *ve* and *sc*). Additionally, *Calventer* **n. g.** is close to genera *Neoprothrix* and *Neopropilus* (Sierraphytoptinae: Sierraphytoptini); however, these two genera retain tibial solenidion *φ* I and opisthosomal seta *c1* (both are absent in *Calventer* **n. g.**).

**Type species:** *Calventer arengii* **n. sp.** (Figure 1, Supplementary Materials SM1), the only currently known species of the genus.

**Host plants and distribution**. Up to now, mites of the genus *Calventer***n. g.** have been recorded only once as vagrants on fronds of *Arenga westerhoutii* Griff. (Arecaceae) in northeastern Vietnam.

**Etymology.** The generic name, *Calventer*, is derived from two words: “calvus”, indicating the absence of the opisthosomal setae *d* and *e*, and “venter”, indicating the reduced chaetom of ventral opisthosoma; gender masculine.

***Calventer arengii* n. sp.** Holotype and paratype females (n = 7). Body flattened, whitish, live mites covered with wax, 171 (171–175), 65 (64–69) wide at the level of setae *c2*. **Prodorsal shield** subtriangular, 45 (41–46) including frontal lobe, 62 (58–63) wide. Frontal lobe 10 (8–11) long, 14 (14–15) wide basally, subtriangular. Cuticle of prodorsal shield with numerous irregularly distributed micropores. Median line weak, consisting of two short fragments in anterior and posterior 1/3 of the shield. Admedian lines distinct, broken, fragmented, forming a central figure consisting of three segments: subhexagonal median segment and suboval anterior and posterior segments. Short thin lines (possible traces of submedian line) present laterally to each of admedians. Prodorsal shield setae *ve* and *sc* thick and stout, *ve* 15 (14–17), directed anterolaterad, tubercles of *ve* situated slightly below anterolateral margin of prodorsal shield, 26 (25–27) apart; *sc* 15 (15–16), 44 (43–46) apart, directed up and divergently anteriad. Epicoxal area with thin smooth ridges.

**Gnathosoma** directed obliquely down and forward. Palps 26 (24–27); chelicerae 22 (20–22). Gnathosomal setae: seta *ν* 1 (1–2); pedipalp genual seta pp*d* angled, 7 (6–7); pedipalp coxal seta *ep* 2 (2–3). Suboral plate subtriangular, smooth, 10 (9–11) long, 20 (20–22) wide.

**Leg I** 37 (36–38), tarsus 7 (7–8), *u′* 5 (5–6), *ft′* 12 (11–14), *ft″* 21 (20–23), *ω* 6 (5–6) knobbed; empodium 6 (5–6), symmetrical, 9-rayed; tibia 10 (9–11), *l′* and *φ* absent; genu 5 (4–5), *l″* 32 (32–35), located on dorsal surface close to tibiogenual articulation; femur 12 (12–14), femoral setae *bv* 14 (14–16).

**Leg II** 33 (33–36), tarsus 7 (7–8), u′ 5 (4–5), ft′ 7 (6–8), ft″ 23 (22–24), ω 6 (5–6) knobbed; empodium 6 (5–6), symmetrical, 9-rayed; tibia 9 (8–9); genu 5 (4–5), l″28 (27–29), located on dorsal surface of genu close to femorogenual articulation; femur 12 (11–13), *bv* 14 (13–15).

**Coxal plates** with irregular short curved cuticular ridges; prosternal apodeme entire, distinct, 9 (8–10). Anterior setae on coxisternum I *1b* 9 (9–10), 14 (14–15) apart; distinct arc-shaped ridge present between tubercles of *1b* and *1a*, proximal setae on coxisternum I *1a* 23 (23–26), 12 (12–14) apart; proximal setae on coxisternum II *2a* 37 (35–41), 31 (29–32) apart; 7 (5–7) narrow smooth coxigenital semiannuli before epigynium.

**External genitalia.** Genital coverflap apically rounded, smooth, basally with tiny microtubercles, 9 (8–10), 20 (19–21), wide; setae *3a* 15 (15–19), 16 (16–18) apart.

**Internal genitalia (n = 3).** Spermatheca tear drop-shaped, 7–8 long, 3–4 wide, directed laterad; spermathecal tubes proximally swollen, narrowed distally, 10–12 long, directed anteriad, located subparallel to longitudinal bridge; spermathecal process non-apparent; longitudinal bridge 16–20; anterior genital apodeme subtrapezoidal, 7–8 long, 19–21 wide basally, 4–5 wide distally.

**Opisthosoma** with 17 (16–18) dorsal annuli and 35 (33–38) ventral annuli between coxa II and caudal lobes. Dorsal annuli form two distinct lateral ridges marked by sharp subtriangular spines. Distal margin of first dorsal annulus forms a plate overlapping with the second dorsal annulus. Microtubercles on ventral annuli absent in anterior one third of opisthosoma, very small behind genital area, becoming larger and pointed beyond setae *c2*, and more elongated and ridge-like on ventral annuli beyond setae *f*. Setal lengths: *c1* absent, *c2* 34 (31–35), *d* and *e* absent, *f* 33 (32–35); *h1* absent; *h2* 38 (37–44); 5 (5–6) ventral annuli from rear shield margin to *c2*; 31 (29–33) annuli between *c2* and *f*; and 4 (4–5) annuli between *f* and *h2*.

**MALE (n = 2).** Body flattened, whitish, 155–162 long, 52–57 wide. Prodorsal shield ornamentation similar to that of female, *ve* 10–12, *sc* 13–14. Genital area subelliptical, *3a* 10–13 long, 10–11 apart, eugenital setae *eu* about 0.5 long. Opisthosoma with 15–16 dorsal and 32–34 ventral annuli with microtubercles similar to that of female; 10–12 annuli between coxae and external genitalia.

**GenBank data.** COI, 1199 bp (Supplementary Materials SM2).

**Host plant.***Arenga westerhoutii* Griff. (Arecaceae: Coryphyoideae: Caryoteae).

**Figure 2:**
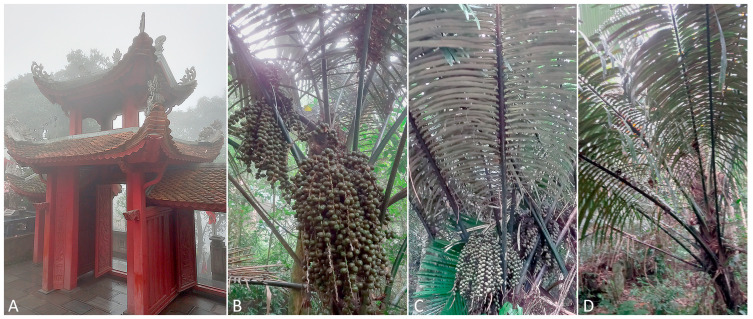
Three sampled specimens of *Arenga westerhoutii* (**B**,**C**,**D**) in the type locality of *Calventer arengii* **n. sp.** near a Buddhist temple (**A**) in Tam Dao Tay Thien in the northeastern Vietnam.

**Relation to the host plant.** All mites were found along the veins on the lower surfaces of old fronds collected from three closely growing palm specimens (Figure 2). No visible damages of fronds were observed.

**Type material.** Type female in slide V81-1, six paratype females, and two paratype males in slides V81-2, V79-1, V79-2 collected in VIETNAM: FJG6+53R Tam Đảo District, near Tam Dao Tay Thien, 21°28′31.7″ N 105°36′36.5″ E, 14 March 2024, coll. P.E. Chetverikov. Type material is kept in Acarological collection of ZIN RAS.

**Etymology**. The species name, *arengii*, is a noun in genitive case, derived from the generic name of the host plant, *Arenga*.

### 3.2. Molecular Phylogenetics

**Figure 3:**
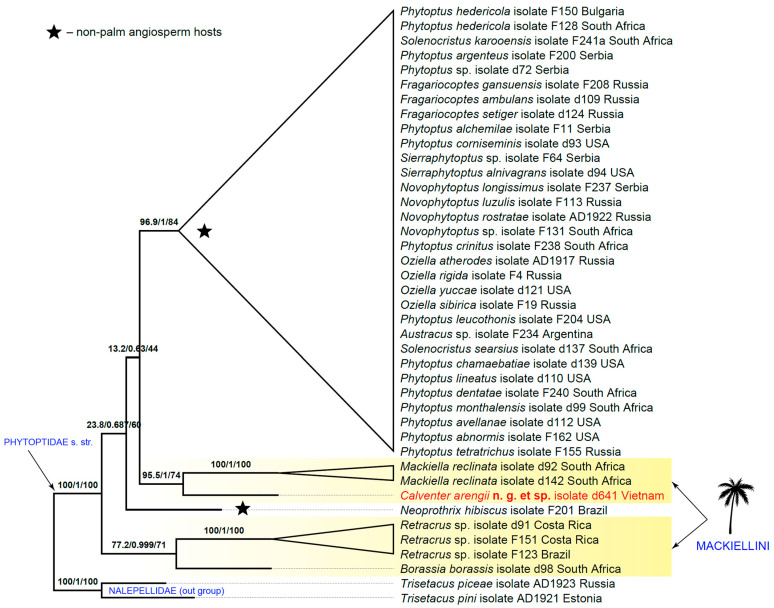
Maximum likelihood *COI* (codons) phylogeny of Phytoptidae *s.str.* showing phylogenetic position of *Calventer arengii* **n. sp.** (colored red) among phytoptid lineages. Branch labels are SH aLRT support (%)/aBayes/UFboot support (%).

All analyses inferred Phytoptidae *s. str.* as comprising a large, moderately supported clade of taxa associated with non-palm angiosperm hosts (eudicots, early-diverging angiosperms, and monocots of the orders Poales and Asparagales) and a basal grade that included mackielline genera from palms and a single taxon, *Neoprothrix hibiscus Reis and Navia*, from eudicots. The latter was previously identified as a rogue taxon contributing to the poor resolution of the phytoptid tree [11]. Both the codon (Figure 3) and amino acid trees indicated a sister relationship between *Mackiella* and *Calventer* **n. g.**, which together formed a moderately supported clade. In contrast, the nucleotide tree resulted in a notably less resolved phylogeny, recovering a weakly supported clade structured as ((*Mackiella*),(*Calventer*, *Borassia*)). Finally, all analyses inferred *Borassia* as grouping with mackielline taxa rather than with phytoptines (*Phytoptus* and *Oziella* in the dataset), contradicting the previous morphology-based transfer of *Borassia* [15] from Mackiellini (Phytoptidae: Sierraphytoptinae) to Phytoptini (Phytoptidae: Phytoptinae).

## 4. Discussion

**Chaetom reduction trends in Eriophyoidea and setal generic formula.** The reliable separation of genera requires distinct discriminative traits that allow the unambiguous assignment of species to a genus. Among various traits used in the current systematics of Eriophyoidea [23], the chaetom of a mite is the primary characteristic for correct generic identification. Plesiomorphically, females of gall mites possess 65 (5 + 6 + 18 + 14 + 8 + 14) setae: 5 prodorsal shield setae (unpaired *vi* and paired *ve* and *sc*), 3 setae on each of the two palps (*v*, pedipalp *d* (=pp*d*) and *ep*), 9 setae on each of the two legs I (*u*′ I, *ft*′ I, *ft*″ I, *emp* I, *ω*, *φ* I, *l*′ I, *l*″ I, and bv I), 7 setae on each of the two legs II (*u*′ I, *ft*′ I, *ft*″ I, *emp* I, *ω*, *l*″ I, and *bv* I), 8 coxigenital setae (paired *1a*, *1b*, *2a*, and *3a*), and 14 opisthosomal setae (paired *c1*, *c2*, *d*, *e*, *f*, *h1* and *h2*); in addition to the listed setae, males have paired setae *eu* situated behind the genital opening [1]. Most of these setae are subject to homoplastic reduction in different lineages of Eriophyoidea; however, some setae (*h2*, *3a*, empodium I and II, tarsal solenidion *ω* I and II) are very stable and present in all known eriophyoids [1,8]. Excluding these “stable” setae, the remaining chaetom of a selected eriophyoid taxon can be represented as a “setal formula”—a table indicating the presence or absence of certain setae (Table 1). The inclusion of such formulas in the descriptions of new genera of Eriophyoidea may be useful for facilitating rapid morphological generic delimitation.

In general, setal reduction is notably more common in Eriophyidae *s. l.* than in Phytoptidae *s. str.* and Nalepellidae, which may reflect both the evolutionary conservatism of the latter two clades and their lower diversity. In this paper, a new palm-associated phytoptid taxon, *Calventer* **n. g.**, has been described from Vietnam. This genus has the most reduced chaetom in the tribe Mackiellini and exemplifies the general setal reduction trend observed in different lineages of Eriophyoidea [1]. Only two phytoptid genera of the tribe Sierraphytoptini, *Neoprothrix* and *Neopropilus*, have a ventral opisthosomal chaetom as reduced as that of *Calventer* **n. g.** Members of all three of these monotypic genera are rare, small-sized vagrant mites associated with exotic host plants that require a warm, wet climate. Although a specific count has not been performed, a rapid survey suggests that eriophyoid taxa with a greatly reduced chaetom are apparently much rarer in the planet’s colder regions. Future studies will help to understand whether this reflects their association with host groups that evolved in biomes near the tropics and equator or their lower resistance to colder climates.

**Problematic phylogeny of Mackiellini.** Contrary to a previously published molecular phylogeny of Phytoptidae *s. str.* [11], the new trees obtained in this study showed all included palm-inhabiting phytoptid genera (*Borassia*, *Calventer* **n. g.**, *Mackiella*, and *Retracrus*) forming a basal grade. Interestingly, our analyses support classifying *Borassia* within Mackiellini (Phytoptinae), reinforcing the older interpretation based on the loss of opisthosomal setae *c1* and its association with palms [24]. This contradicts the recent transfer of *Borassia* to Phytoptinae, which was based on the absence of the dorso-ventral differentiation of the opisthosomal annuli and a worm-shaped body [15].

Although no analysis to date has inferred the monophyly of Mackiellini, the inclusion of *Calventer* **n. g.** notably brought the mackiellines closer together in the tree. If this is a true tendency, a future increase in the number of phytoptid genera in the dataset may potentially result in a monophyletic Mackiellini. This tribe was previously hypothesized to be an early branch of phytoptids that evolved on palms [25,26], which are themselves an exceptionally ancient monocot lineage [27]. Morphologically, mackiellines are united by the reduction in opisthosomal setae *c1* and, considering their distinct host associations, may represent a true early-diverged clade of Phytoptidae *s. str.* The future collection of rarely encountered phytoptid genera such as *Acathrix*, *Neopropilus*, *Neoprothrix*, and *Propilus*, alongside an investigation into their morphology including a comparative anatomy of the spermathecal apparatus and sequencing, will help test this hypothesis. Additionally, the systematic position of another mackielline genus, *Palmiphytoptus*, needs to be tested. This genus lacks any distinct character typical for Phytoptidae besides the anterior position of the single pair of prodorsal shield setae (putative *ve*), which may in fact be *sc* displaced anteriorly (in which case, the genus should belong to Eriophyidae) [8].

Our estimates of the phylogenetic position of *Calventer* **n. g.** suggest the palm-inhabiting genus *Mackiella* to be its most probable sister taxon. However, considering the incomplete set of phytoptid genera available for analysis, this estimate requires additional testing. Remarkably, in our analyses, the genus *Neoprothrix* (Sierraphytoptini) consistently tended to occupy a position in the basal part of the phytoptid tree, close to the clades containing mackiellines. This monotypic genus was initially described as the only eriophyoid taxon possessing paired setae *vi* [27]; however, morphological reinvestigation with confocal laser scanning microscopy (CLSM) revealed that the structures originally identified as *vi* are two internal rod-like apodemes [28]. Similarly to *Calventer* **n. g.**, *Neoprothrix* lacks opisthosomal setae *d* and *e*; however, whether this loss is a synapomorphy or a homoplasy remains unknown. *Neoprothrix* is associated with the eudicot host *Hibiscus* (eudicots: Malvales: Malvaceae), while *Calventer* **n. g.**, like all mackiellines, inhabits palms (monocots: Arecales: Arecaceae). This host difference weakens the putative relationship between *Calventer* **n. g.** and *Neoprothrix*. However, there is evidence that mackiellines are capable of significant host shifts to phylogenetically distant hosts. For instance, two species of the mackielline genus *Retracrus* were described from Brazil on heliconias (monocots: Zingiberales: Heliconiaceae), while the rest of the *Retracrus* spp. are associated with palms [29,30]. Overall, the available data are insufficient to resolve the phylogeny of Mackiellini and indicate that host shifts are a major complicating factor in reconstructing Phytoptidae *s. str.* relationships.

**Are phytoptids rare in Asia?** The global distribution of recorded species and newly described taxa within Phytoptidae *s. str.* is heavily skewed, with the majority of data originating from Europe and the Americas. In contrast, Asia and Africa are notably under-represented, while Australasia remains virtually unexplored. This bias reflects the historical and recent intensity of phytoptid research in regions like the USA, Brazil, and Russia. The foundational works of H. Keifer (1902–1986), complemented by modern PhD theses from D. Navia (Brazil) and P. Chetverikov (Russia), have yielded extensive publications on phytoptids from palms, sedges, and other angiosperms [11,26,29,30,31,32]. This pattern is a classic example of a “collector effect,” where a taxon’s observed diversity sharply declines in under-sampled regions—a phenomenon previously noted in eriophyoid studies [33]. This geographical bias is critical when evaluating broad biogeographic claims. Ozman-Sullivan and Sullivan [34,35] have challenged recent analyses [36,37], which concluded that eriophyoid mite richness and endemism peak in temperate zones—a pattern inverse to that of their host plants. They argue that such conclusions are likely an artifact of uneven sampling.

The current knowledge of Asian mackiellines starkly illustrates this point: it is limited to a single record of *Borassia* from India [24] and the new genus *Calventer* described here from Vietnam. This scarcity of records almost certainly does not reflect a true absence; a rich mackielline fauna is expected to exist on the diverse Arecaceae of Asia and Australasia, awaiting discovery. Furthermore, given the known association of many phytoptids with superrosid plants and their tendency to colonize endemic and relict hosts [11], targeted surveys in Asia on key orders (e. g., Rosales, Fabales, Fagales) and endemic eudicots and monocots will undoubtedly reveal a much greater diversity of Phytoptidae *s. str.*, reshaping our understanding of their true distribution and evolution.

## Figures and Tables

**Table 1:** Setal formulas (presence “+” or absence “−”) for seven palm-inhabiting phytoptid genera.

Genus	*vi*	*ve*	*sc*	*ppd*	*c1*	*c2*	*d*	*e*	*f*	*h1*	*1a*	*1b*	*2a*	*bv* I/II	*l*″ I/II	*l*′ I	φ I	*ft*′ I/II	*ft*″ I/II	*u*′ I/II
*Acathrix* Keifer 1962	−	+	+	+	+	+	+	+	+	+	+	+	+	+/+	+/+	+	+	+/+	+/+	+/+
*Borassia* Chetverikov, Craemer 2017	−	+	+	+	−	+	+	+	+	+	+	+	+	+/+	+/+	+	−	+/+	+/+	+/+
*Calventer* **n. g.**	−	+	+	+	−	+	−	−	+	−	+	+	+	+/+	+/+	−	−	+/+	+/+	+/+
*Mackiella* Keifer 1939	−	+	+	+	−	+	+	+	+	+	+	+	+	+/+	+/+	+	+	+/+	+/+	+/+
*Palmiphytoptus* Navia & Flechtmann 2002	−	+	−	+	−	+	+	+	+	+	+	+	+	+/+	+/+	−	−	+/+	+/+	+/+
*Propilus* Keifer 1975	−	+	−	+	−	+	+	+	+	+	+	+	+	+/+	+/+	−	−	+/+	+/+	+/+
*Retracrus* Keifer 1965	−	+	+	+	−	+	+	+	+	−	+	+	+	+/+	+/+	+	+	+/+	+/+	+/+

## Data Availability

The original contributions presented in this study are included in the article and Supplementary Materials (SM1 and SM2). Further inquiries can be directed to the corresponding author.

## Supplementary Materials

The following supporting information can be downloaded at: https://www.mdpi.com/article/10.3390/insects16111113/s1, SM1: PC LM microphotographs of *Calventer arengii* n. sp.; SM2: *COI* partial sequence of *C. arengii* n. sp.
