# Supplementary material for: A New Genus of Four-Legged Mites from Palms in Vietnam: The Morphology and Phylogeny of Calventer arengii n. g. & sp. (Eriophyoidea, Phytoptidae)
Source: Insects. 2025 Oct 31;16(11):1113. doi: 10.3390/insects16111113 (PMC12653530; doi:10.3390/insects16111113)
Supplement: Supplementary file 1 [file insects-16-01113-s001.zip › SM1_photographs.pdf]

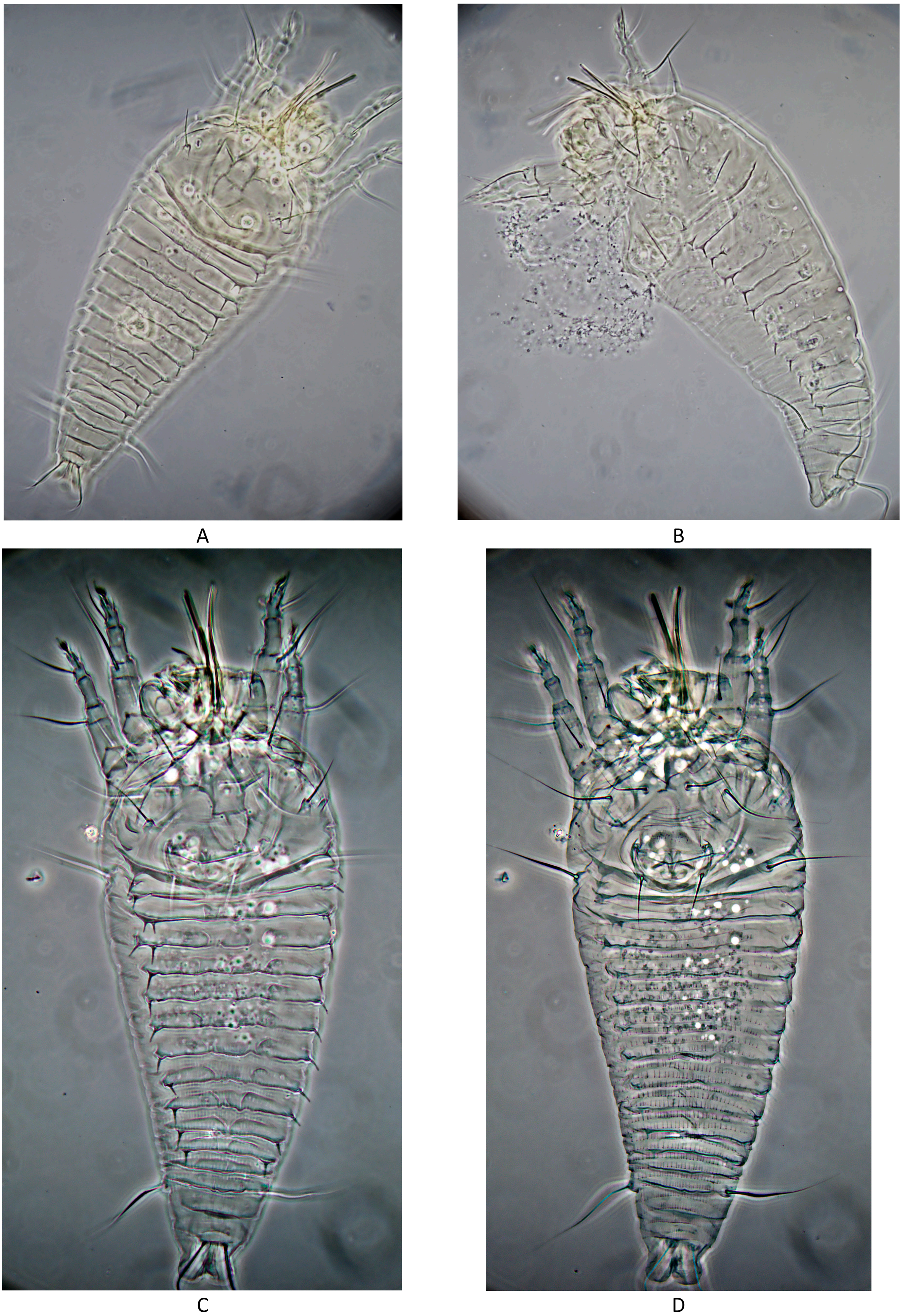

Figure S1. PC LM microphotographs of *Calventer arengii* n. sp. A – dorsal view of female, magnification x400; B – lateral view of female, magnification x400; C – dorsal view of female, magnification x1000; D – ventral view of female, magnification x1000.
