# Supplementary material for: A New Genus of Four-Legged Mites from Palms in Vietnam: The Morphology and Phylogeny of Calventer arengii n. g. & sp. (Eriophyoidea, Phytoptidae)
Source: Insects. 2025 Oct 31;16(11):1113. doi: 10.3390/insects16111113 (PMC12653530; doi:10.3390/insects16111113)
Supplement: Supplementary file 1 [file insects-16-01113-s001.zip › SM2_COI_seq.pdf]

>Calventer_arengii_COI_partial_sequence

**TGTATTTATTTTTTGGATTTTGGTCTTCTTTATATGGTACTTCTTTGAGGTTTTTTATTCGTCTTGAGCTTTCAACTAGTGGATCT
TTTGTTTTTAATGATCATATTTATAATGTTTTAGTTACTTCTCATGCTTTTGTTATAATTTTTTTTGTTGTTATGCCTATTTTAATA
GGGGGGTTTGGTAATTGACTTCTTCCTCTTTTATTGGGTTGTAATGATATAGCTTTTCCTCGAATAAATAATTTGAGTTTTTGA
CTTTTAGTACCTTCTATGTTTTTTTTATTTGGTTCTTTTTTTTTTTTTTCTGGTGCTGGAACTGGGTGAACTGTTTATCCTCCTCTT
TCTTCTTTTTTTTATCATTCTGATGGTTCTGTAGATTTGTTATTTTTTCTTTACATATTGGTGGAGTTTCTTCTATTTTGAGATCT
ATTAATTTTATTAGAACTATTTTTACTTTTCGTTCTTTTGGAATTTTTATGGATAAGTTAGATTTGTTTGTTTGGTCTATTTTAGT
TACTTCATTTCTTTTGCTTTTTTCATTACCTGTATTAGCAGGTTGTATTACAATGCTTCTTACTGATCGTAATTTTAATACTTCTT
TTTTTGATCCTTTAGGAGGAGGTGATCCTATTCTTTATCAACATTTATTTTGGTTTTTTGGTCATCCTGAGGTTTATATTTTAAT
TCTTCCTGGTTTTGGTATTATTTCTCATGTTTTGTCTTTTTATTGTGGTAAGTCTGAGCCTTTTGGTTATATGGGTATGGTTTAT
GCTATTATTTCTATTGGTTTATTGGGATTTGTTGTATGGGCTCATCATATGTTTACCGTTGGTATAGATGTTGATACTCGTGCT
TATTTTACTTCTGCTACTATGATTATTGGAGTTCCTACTGGAATTAAGATCTTTAGTTGGTTATCTACTATCTTATTTTCTAATTT
GAGGTTCGATGTTCCATTTTATTGGTGTTTAGGTTTTATTTTTTTATTTACTTTGGGTGGTTTGACTGGTATTGTTTTGTCTAAT
TCATCTTTGGATATTGTTCTCCATGATACTTACTACGTTGTAGCTCATTTTCATTATGTTCTTTCTATAGGAGCTGTTTTTTCTAT
TTTTGCTGGATTTTTGCATTGGTTTAATATTTTTTATGGTTATAGTCTTTCTTCATTTGCTTTGAAGATTCATTTTTTAGTTCTT**

An official website of the United States government Here's how you know

NIH National Library of Medicine
National Center for Biotechnology Information

Log in

Notice

Because of a lapse in government funding, the information on this website may not be up to date, transactions submitted via the website may not be processed, and the agency may not be able to respond to inquiries until appropriations are enacted. The NIH Clinical Center (the research hospital of NIH) is open. For more details about its operating status, please visit cc.nih.gov. Updates regarding government operating status and resumption of normal operations can be found at opm.gov.

```
LOCUS       Seq1                    1199 bp    DNA     linear       07-OCT-2025
DEFINITION  Phytoptidae gen. sp. isolate d641 mitochondrion.
ACCESSION
VERSION
KEYWORDS    .
SOURCE      mitochondrion Phytoptidae gen. sp.
  ORGANISM  Phytoptidae gen. sp.
            Unclassified.
REFERENCE   1  (bases 1 to 1199)
  AUTHORS   Chetverikov,P.E.
  TITLE     A New Genus of Four-legged Mites from Palms in Vietnam: Morphology
            and Phylogeny of Calventer arengii n. gen. et sp. (Eriophyoidea,
            Phytoptidae)
  JOURNAL   Unpublished
REFERENCE   2  (bases 1 to 1199)
  AUTHORS   Chetverikov,P.E.
  TITLE     Direct Submission
  JOURNAL   Submitted (07-OCT-2025) Parasitology, Zoological Institute of RAS,
            Universitetskaya nab. 1, Saint-Petersburg 199034, Russia
COMMENT     ##Assembly-Data-START##
            Sequencing Technology :: Sanger dideoxy sequencing
            ##Assembly-Data-END##
FEATURES             Location/Qualifiers
     source          1..1199
                     /organism="Phytoptidae gen. sp."
                     /organelle="mitochondrion"
                     /mol_type="genomic DNA"
                     /isolate="d641"
                     /geo_loc_name="Viet Nam"
                     /collection_date="14-Mar-2024"
BASE COUNT      214 a    140 c    203 g    642 t
ORIGIN
        1 tgtatttatt ttttggattt tggtcttctt tatatggtac ttctttgagg ttttttattc
       61 gtcttgagct ttcaactagt ggatcttttg tttttaatga tcatatttat aatgttttag
      121 ttacttctca tgcttttgtt ataatttttt ttgttgttat gcctatttta ataggggggt
      181 ttggtaattg acttcttcct cttttattgg gttgtaatga tatagctttt cctcgaataa
      241 ataatttgag tttttgactt ttagtacctt ctatgttttt tttatttggt tctttttttt
      301 ttttttctgg tgctggaact gggtgaactg tttatcctcc tctttcttct tttttttatc
      361 attctgatgg ttctgtagat tttgttattt tttctttaca tattggtgga gtttcttcta
      421 ttttgagatc tattaatttt attagaacta tttttacttt tcgttctttt ggaattttta
      481 tggataagtt agatttgttt gtttggtcta ttttagttac ttcatttctt ttgctttttt
      541 cattacctgt attagcaggt tgtattacaa tgcttcttac tgatcgtaat tttaatactt
      601 ctttttttga tcctttagga ggaggtgatc ctattcttta tcaacattta ttttggtttt
      661 ttggtcatcc tgaggtttat attttaattc ttcctggttt tggtattatt tctcatgttt
      721 tgtcttttta ttgtggtaag tctgagcctt ttggttatat gggtatggtt tatgctatta
      781 tttctattgg tttattggga tttgttgtat gggctcatca tatgtttacc gttggtatag
      841 atgttgatac tcgtgcttat tttacttctg ctactatgat tattggagtt cctactggaa
      901 ttaagatctt tagttggtta tctactatct tattttctaa tttgaggttc gatgttccat
      961 tttattggtg tttaggtttt atttttttat ttactttggg tggtttgact ggtattgttt
     1021 tgtctaattc atctttggat attgttctcc atgatactta ctacgttgta gctcattttc
     1081 attatgttct ttctatagga gctgtttttt ctatttttgc tggatttttg cattggttta
     1141 atatttttta tggttatagt ctttcttcat ttgctttgaa gattcatttt ttagttctt
```
